# Uptake and physiological impacts of nanoplastics in trees with divergent water use strategies†

**DOI:** 10.1039/d4en00286e

**Published:** 2024-07-11

**Authors:** Maria Elvira Murazzi, Alice Pradel, Roman B. Schefer, Arthur Gessler, Denise M. Mitrano

**Affiliations:** a WSL Swiss Federal Institute for Forest, Snow and Landscape Research Zürcherstrasse 111 8903 Birmensdorf Switzerland; b Department of Environmental Systems Science, ETH Zurich Universitätstrasse 16 8092 Zurich Switzerland Denise.mitrano@usys.ethz.ch

## Abstract

Anthropogenic contaminants can place significant stress on vegetation, especially when they are taken up into plants. Plastic pollution, including nanoplastics (NPs), could be detrimental to tree functioning, by causing, for example, oxidative stress or reducing photosynthesis. While a number of studies have explored the capacity of plants to take up NPs, few have simultaneously assessed the functional damage due to particulate matter uptake. To quantify NPs uptake by tree roots and to determine whether this resulted in subsequent physiological damage, we exposed the roots of two tree species with different water use strategies in hydroponic cultures to two concentrations (10 mg L^−1^ and 30 mg L^−1^) of model metal-doped polystyrene NPs. This approach allowed us to accurately quantify low concentrations of NPs in tissues using standard approaches for metal analysis. The two contrasting tree species included Norway spruce (*Picea abies* [L.] Karst), a water conservative tree, and wild service tree (*Sorbus torminalis* [L.] Crantz), an early successional tree with a rather water spending strategy. At both exposure concentrations and at each of the experimental time points (two and four weeks), NPs were highly associated and/or concentrated inside the tree roots. In both species, maximum concentrations were observed after 2 weeks in the roots of the high concentration (HC) treatment (spruce: 2512 ± 304 μg NPs per g DW (dry weight), wild service tree: 1190 ± 823 μg NPs per g DW). In the aboveground organs (stems and leaves or needles), concentrations were one to two orders of magnitude lower than in the roots. Despite relatively similar NPs concentrations in the tree aboveground organs across treatments, there were different temporal impacts on tree physiology of the given species. Photosynthetic efficiency was reduced faster (after 2 weeks of NPs exposure) and more intensively (by 28% in the HC treatment) in wild service trees compared to Norway spruce (*ca.* 10% reduction only after 4 weeks). Our study shows that both, evergreen coniferous as well as deciduous broadleaf tree species are negatively affected in their photosynthesis by NPs uptake and transport to aboveground organs. Given the likelihood of trees facing multiple, concurrent stressors from anthropogenic pollution and climate change, including the impact of NPs, it is crucial to consider the cumulative effects on vegetation in future.

## Abstract 2

Anthropogenic contaminants can place significant stress on vegetation, especially when they are taken up into plants.

## Graphical abstract

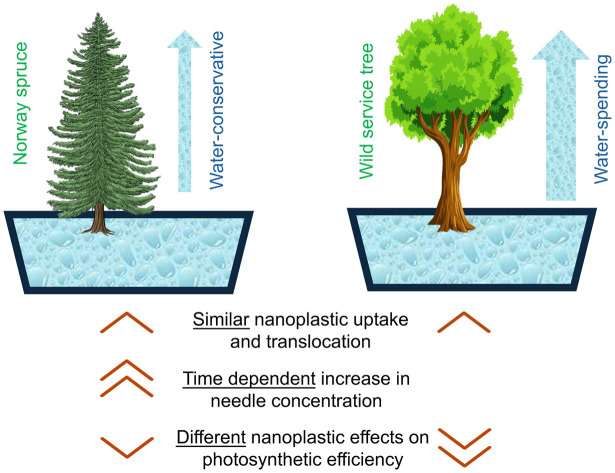

**Environmental significance**

Plants, including trees, have been shown to uptake nanoplastics but less information is known regarding extent of uptake depending on physiology. We show that nanoplastics can be transported from roots to above ground tree organs and impacts of this pollution can vary based on factors such as tree physiology, needle morphology, and water use strategies. In conjunction with other stressors from anthropogenic pollution and climate change which will impact vegetation, nanoplastics can alter their physiological functioning.

## 1. Introduction

Urbanization increases local pollution from a variety of anthropogenic sources. This reduces air, water and soil quality, which in turn adds pressure on urban green spaces.^1^ Plastic pollution, including nanoplastics (NPs, particles <1 μm in size), adds to this burden of anthropogenic contaminants in highly populated zones and could place additional stresses on local vegetation. The myriad of plastics found in cities originating from different sources, ranging from construction sites to mismanaged plastic litter, can eventually fragment into NPs and be accumulated in urban waters and soils.^2^ Additionally, like other airborne contaminants, NPs can be washed out from the atmosphere,^3^ thus moving them from the air into urban soils. Greening cities through blue/green infrastructure projects has been one approach to improve urban air and water quality. Urban tress, for example, have been shown to help improve air quality by facilitating widespread deposition of various gases and particles through the provision of large surface areas as well as water quality and management by minimizing flooding.^4,5^ Currently, most studies focus on the capacity of plants and other biological media to adsorb airborne particulate matter or to bioconcentrate pollutants in remediation efforts,^6,7^ but few explore the functional damage caused by particulate matter uptake from air, soil, or water by urban vegetation.

Nanoplastics have been shown to be taken up by plant roots and transported to other plant organs.^8–14^ While the entry mechanisms into the roots are not entirely clear to date, Li *et al.* hypothesised that NPs follow a crack entry mode through discontinuous regions in the Casparian strip.^9^ The Casparian strip is made up by the microscopically visible thickened and suberized cell walls of the root endodermis. It renders the cell walls relatively impermeable to water and thus normally presents a barrier to inward movement of water and solutes in the apoplast and forces transport through the selective plasma membrane into the cytoplasm of the living cells of the symplast.^15^ It could be envisioned that high transpiration rates increase NPs uptake and transport to the shoots of plants because of increased water fluxes when transport occurs mainly *via* crack entry on the apoplast route in regions where the Casparian strip is not fully developed (root tips and initiation zone of secondary roots). If transport occurs mainly through cells, as forced by the intact Casparian strip, however, there would not necessarily be a proportional relationship between water flow and particle uptake. Here, transmembrane transport processes would most likely decouple NPs uptake and transport from tree water fluxes. In trees, Murazzi *et al.* observed transport of NPs in an early successional water spending species (*Betula pendula*) to the stem, whereas in more water conservative late successional species (*Quercus petraea*, *Picea abies*), no such allocations could be detected.^16^ This also supports the hypothesis that the water mass flow through the plant and thus the crack entry mode might be responsible for particle uptake. However, the method for detecting NPs in plant tissues used by Murazzi *et al.* was not highly sensitive, thus more work is needed to better understand how water use strategies of tree species affect NPs uptake and transport in trees.

Furthermore, it remains unclear if and how uptake of NPs affects the functioning and physiology of plants. In general, knowledge on the impact of NPs on plant functioning, including systemic effects, is limited^17^ and there are few studies in trees specifically. One further confounding factor in assessing organism response to NPs is that many commercially available model NPs contain a biocide to preserve the particles,^18^ but this is often not tested for and reported in the context of effects studies. In maize seedlings, it was observed that after 15 days of polystyrene NPs exposure, root primary metabolism was altered and the root antioxidative system was upregulated whereas photosynthetic functioning (as indicated by optimum photosynthetic quantum yield (*F*_v_/*F*_m_)) was not affected.^19^ In contrast, polymethyl methacrylate NPs exposure to roots led to a reduced photosynthetic capacity in *Lactuca sativa*, indicating a systemic response. Chlorophyll fluorescence has been shown to be a highly sensitive method to analyze photosynthetic activity and to study its systemic responses to various stressors, including pathogens and herbivores,^20^ heat and mechanical damage,^21^ drought^22^ and toxic contaminants.^23^ Thus, if any systemic effects due to NPs occurs which either affects photosynthesis directly or indirectly *via e.g.*, phytohormonal actions or changes in root metabolic activity, changes in water, carbon or nutrient transport,^24^ the change in chlorophyll fluorescence is amongst the most sensitive indicators of impaired photosynthetic activity.

If any part or component of the light or dark reaction of photosynthesis is impaired, excessive light energy that cannot be used for the production of chemical energy (ATP, NADH/H^+^, carbohydrates) could cause light damage by photooxidation.^25^ To prevent such damage, plants have evolved photoprotection mechanisms where mainly carotenoids play a role in quenching excessive light energy by dissipating it to heat.^26^ Thermal energy dissipation under excess light is accomplished in the xanthophyll cycle *via* the de-epoxidation of violaxanthin resulting in the production of zeaxanthin.^27^ Assessing the reflectance of leaves at wave-lengths sensitive to pigment composition and absorption changes provides a non-destructive technique to obtain insight into such pigment conversion processes. The photochemical reflectance index (PRI) that is derived from a narrow-band reflectance at 531 and 570 nm is a measure for photosynthetic radiation use efficiency. It is related to the conversion of violaxanthin to zeaxanthin on the short term^28^ and on the longer term, to the relative change of carotenoids compared to chlorophyll.^29^ Thus, measurements of chlorophyll fluorescence and PRI provide complementary information on the efficiency of photosynthesis and on the impairment of that efficiency under stress.

To assess if NPs were taken up by tree roots and transported to aboveground plant organs, we exposed the roots of two tree species with different transpiration mechanisms in hydroponic cultures to two concentrations (low and high) of model polystyrene NPs. Here, quantification of trace concentrations of NPs in the trees were facilitated by a palladium dopant incorporated during NPs synthesis, using the metal as a proxy for the plastic. The two contrasting tree species included a water conservative late successional, shade tolerant species with xeromorphic needle traits (Norway spruce; *Picea abies* (L.) H. Karst.) and a light demanding, thermophilic species with a water spending strategy and low drought tolerance (wild service tree, *Sorbus torminalis* (L.) Crantz).^30^ Stomatal conductance is under non-restricted water availability approx. 10-fold lower in Norway spruce seedlings compared to wild service trees^30,31^ leading to transpiration rates being an order of magnitude lower in the coniferous spruce compared to the broadleaf species. To assess if systemic impacts of NPs uptake and transport occurred, we determined potential quantum yield of photosystem II (*F*_v_/*F*_m_, where *F*_v_ is the variable and *F*_m_ the maximal fluorescence), PRI and additional reflectance indices sensitive to carotenoids. We hypothesized that the less water conservative species with known high transpiration rates (wild service trees) would take up NPs faster compared to xeromorphic Norway spruce. This is because the assumed uptake mode of NPs *via* a crack entry mode would lead to higher particle influx and transport within the plants with higher water use. Additionally, we hypothesized that particle uptake *via* the roots and transport to the leaves or needles could lead to systemic impairment of tree functioning, and that this would be indicated by changes in chlorophyll fluorescence and PRI. Consequently, the damage would be seen earlier in our short-term exposure experiment in the high-water use species, wild service trees. By more clearly understanding not only the uptake dynamics of NPs in trees with contrasting traits, but also the impacts on tree functioning and vitality, a clearer picture can be formed regarding the impacts of increased (nano)plastics pollution. This is especially important given the increased likelihood of NPs exposure in hot spots of urban contamination and in light of urban planners more regularly using trees and other plant species in the context of blue/green infrastructure projects.

## 2. Materials and methods

### 2.1. Model nanoplastics synthesis and characterization

Model palladium (Pd) doped polystyrene nanoplastics (PS-Pd-NP) were synthesized in house following Mitrano *et al.*^32^ The PS-Pd-NP stock suspension was quantified by inductively coupled plasma-mass spectrometry (ICP-MS, Agilent 8900) following acid digestion to assess Pd content (209.3 ± 8.5 mg Pd per L (mean ± SD, *n* = 3)). The Pd content in the NPs corresponds to 0.295 ± 0.009 w/w%. Therefore, the stock solution contained 70.97 g NP per L. Initial particle size and surface charge were measured on a Malvern Zetasizer at 25 °C, where the hydrodynamic was 231.6 ± 60.09 nm and the zeta potential was −39.7 ± 5.28 mV in deionized water. While not explicitly tested in this study, the authors have extensively tested the leaching potential of the Pd tracer from the NPs and have found no appreciable release under a variety of conditions including plant growth media and wheat plants,^11^ biological media including simulated gastrointestinal fluid,^33^ ozonation related to drinking water treatment plants,^34^ and wastewater treatment plants.^35^

To study particle agglomeration in the plant growth media, we incubated PS-Pd-NP in glass beakers filled with either growth media or deionized water (serving as a control) for one week. Four beakers (2 × growth media and 1 × control) contained a low PS-Pd-NP concentration (10 mg L^−1^) and four beakers (3 × growth media and 1 × control) contained a high PS-Pd-NP concentration (30 mg L^−1^). Every two days the solutions underwent aeration (30 minutes) using an aquatic pump, with the collection of two surface aliquots per beaker to assess agglomeration *via* dynamic light scattering (DLS). After one week, the PS-Pd-NP behavior at both low and high PS-Pd-NP concentrations behaved similarly in the growth media where the mean particle size increased on average to 497 ± 135 nm and the zeta potential decreased to −25.3 ± 3.5 mV. In comparison, the size and zeta potential in deionized water remained stable (233.3 ± 69.1 nm and −35.0 ± 6.7 mV). The full time series can be found in Fig. S1.†

### 2.2. Tree cultures and climate chamber experiment protocol

100 seedlings (1-month old) of the deciduous wild service tree (*Sorbus torminalis* [L.] Crantz) and 100 seedlings (1-year old) of the conifer Norway spruce (*Picea abies* [L.] Karst) were brought into a climate chamber (25 °C, 50% of humidity, day/night photoperiod 12/12 h) and potted in 10 cm diameter plastic pots with a mixed soil substrate (“Containererde”, Ökohum GmbH, Switzerland). The seedlings were watered three times a week to field capacity and their position was randomly changed within the climate chamber once per week. After 6 months, 48 wild service trees and 96 Norway spruce were carefully removed from the soil and rinsed with tap water to remove all visible soil particles from their roots. We are aware of the fact that this procedure could have caused some damage to the fine roots, which could have facilitated penetration of NPs into the root system. Batches of 12 trees of the same species were then transferred into a hydroponic growth system. The roots where submersed in an 1/8 Hoagland nutrient solution containing KNO_3_ (0.152 g L^−1^), NH_4_H_2_PO_4_ (0.058 g L^−1^), MgSO_4_–7H_2_O (0.062 g L^−1^) and Ca(NO_3_)_2_–4H_2_O (0.236 g L^−1^), (2.8 L filled in a glass container (Pyrex®, 4 L capacity)). The aboveground parts of the plants were separated from the solution and supported by a floating 1.4 cm thick waterproof ethylene-vinyl acetate mat (Fig. S2†). Plants were inserted at the root crown *via* slots in the mat and remained untouched for the remainder of the exposure period. As we could quantify the PS-Pd-NP exposed to the plants *via* the metal-doping, we could ensure we did not quantify incidental NPs translocation into the plants in the unlikely event that the mat fragmented during the exposure period.

Seedlings were exposed to two concentrations of model PS-Pd-NP added directly to the nutrient solutions, 10 mg L^−1^ (low concentration treatment (LC)) and 30 mg L^−1^ (high concentration treatment (HC)), and to a control nutrient solution (no PS-Pd-NP added). All solutions were renewed every week in the hydroponic chamber over the course of the experiment with the same initial concentration of PS-Pd-NP. Wild service trees were exposed for a total of two weeks while spruce trees were exposed for a duration of either two or four weeks in separate experimental set-ups. Three containers with 12 plants each were attributed to each treatment concentration, exposure time and species combination with a further 6 containers serving as untreated controls (two for each exposure time and species combination). Each container was aerated for half an hour on the first day of the experiment and again every two days until the end of the exposure time to avoid anoxic conditions in the solution and facilitate PS-Pd-NP dispersion in the media.

While hydroponic systems differ from soil substrates, they allow us to determine physiological impacts on trees in response to a specific NPs dose. It has been shown for microplastics^36^ as well as for organic and heavy metal pollutants^37^ that the impact on functioning is different between soil and hydroponically grown plants with often stronger negative effects in hydroponic cultures. This might be due to absence of contaminant adsorption to and retention by the solid matrix in the case of hydroponic systems. However, hydroponic systems provide the advantage of a more defined NPs exposure regime, as otherwise the soil physico-chemical properties could modify the dose the roots are exposed to.

### 2.3. Plant physiology parameters

At the start of the experiment (week 0), three leaves of wild service tree or a bunch of needles of Norway spruce from the top of the crown of three different trees in each container were selected to measure the chlorophyll fluorescence of photosystem II and reflectance indices. Weekly measurements on the same designated leaf or bunch of needles were performed throughout the experimental period. The potential quantum yield of photosystem II (*F*_v_/*F*_m_) was determined with the PAR-FluorPen FP 110-LM (PSI, Drásov, Czech Republic) in 30 min dark adapted leaves. According to Bilger, *et al.*,^38^*F*_v_/*F*_m_ is a sensitive indicator of plant photosynthetic performance and decreases when plants are exposed to various types of stresses.^39^ With the PolyPen RP410 (PSI, Drásov, Czech Republic) several reflectance indices can be determined, including the photochemical reflectance index (PRI). This can track slow changes in the carotenoid/chlorophyll ratio, *e.g.*, over the growing season and/or faster changes in the epoxidation status of the xanthophyll cycle. PRI was calculated as the normalized difference of reflectance at 531 nm (eqn (1)) according to Gamon, *et al.*^40^

1 PRI = (*R*_531_ − *R*_570_)/(*R*_531_ + *R*_570_)

where *R* stands for the reflectance at the wavelength of 531 or 570 nm.

The normalized pigments chlorophyll ratio index (NCPI) was also measured for the carotenoid/chlorophyll ratio^41^ and was calculated following eqn (2):

2 NCPI = (*R*_680_ − *R*_430_)/(*R*_680_ + *R*_430_)

where *R* is the reflectance at the specific wavelengths.

The carotenoid index is a specific proxy for the carotenoid content in leaves and was calculated according to eqn (3)^42^ with *R*_510_ and *R*_550_ indicating the wavelength-specific reflectance:

3 Carotenoid index = 1/*R*_510_ − 1/*R*_550_

### 2.4. Plant harvest

After two or four weeks of exposure, each tree was carefully removed from the hydroponic system. The roots and the root collar section inserted in the waterproof mat that was moistened by the suspension were collected from each tree. These were then washed intensively with deionized water for five minutes, dried with a paper towel and weighed using a precision laboratory balance (PM 200, Mettler-Toledo, Columbus, OH, USA). The leaves or needles were then separated from the stem and both the aboveground tissues were separately weighed. The three tissues of each tree (roots, stems and leaves or needles) were then oven dried at 60 °C for four days. Subsequently the dry weight (DW) was recorded for each tissue.

### 2.5. Sample preparation and ICP-MS analysis

After drying, each of the three different tissues from each tree organ replicate were homogenized individually using a zircon ball mill at a frequency of 30 Hz (MM 400, Retsch, Haan, Germany). To lower the risk of cross-contamination, the mill was thoroughly cleaned before milling each sample, with tissue controls being milled first, followed by the low and high exposure treatments, respectively. Each homogenised sample consisting of the entire tree organ was mixed with 1 mL HNO_3_ and 1 mL H_2_0_2_ and subsequently underwent microwave acid digestion (ultraWAVE microwave digestor from Milestone Srl, Italy), where the temperature and pressure were ramped to 220 °C and 160 bar over 30 minutes, then maintained at 220 °C and 160 bar for 30 minutes and returned to ambient temperature and pressure over approximately 30 minutes. After digestion, each sample was quantitatively transferred to a Falcon tube and diluted with ultrapure water up to a final volume of 10 mL. Subsequently, samples were centrifuged (Allegra X-30R centrifuge from Beckman Coulter, United States) for 10 minutes at 10 000 relative centrifugal force (RCF) to remove residual biomass particulate matter prior to ICP-MS (Agilent 8900) analysis for Pd quantification as a proxy for the NPs concentration. For each measurement, 3900 μL of the sample was transferred to an ICP-MS analysis tube and 100 μL of internal standards (rhodium and indium) were added. Internal standards were monitored over the entire analysis. Limits of detection (LOD) ranged from 0.019 to 0.023 μg Pd per L and limits of quantification (LOQ) ranged from 0.056 to 0.061 μg Pd per L. ICP-MS tuning and Pd calibration curves ranging from 0 to 25 μg L^−1^ were made daily. Internal standards and calibration solutions were purchased from Sigma-Aldrich.

The concentration of PS-Pd-NPs for different exposure times, concentrations, species and tissues were corrected by subtracting the measured Pd per dry mass of biomass in the control treatments for each respective exposure time, species and tissue.

### 2.6. Controls and spiked additions to assess PS-Pd NP recovery from tree biomass

In order to assess the recovery of PS-Pd NP from the tree biomass matrices across our entire analytical workflow, we conducted spiked additions of PS-Pd-NP dispersions (1 μg L^−1^, 2.5 μg L^−1^, and 5 μg L^−1^) to known masses of each dried tissue (roots, stems and leaves or needles) biomass. The content of PS-Pd-NP and of biomass were selected based on the anticipated tree growth and PS-Pd-NP uptake. The spiked samples underwent the same digestion and analysis method as described above. The average recoveries across all tissues for both tree species were approximately 99% (Fig. S3†), indicating we could accurately quantify PS-Pd-NP which were taken up into the tree organs.

### 2.7. Data analysis and statistics

Statistical analysis was performed using RStudio 2022.07.1. Data were tested for normal distribution by applying a Kolmogorov–Smirnov test. For data associated with NPs uptake and translocation, the dataset was divided by tissue type into three subsets: leaves/needles, stems, and roots. For each subset, a linear model examined the effects of concentration, time, and species on NPs translocation. Interactions between concentration–time and concentration–species were included. Estimated marginal means (EMMs) were calculated for the concentration–time interaction using the emmeans package, with pairwise comparisons performed to identify significant differences. Additionally, subsets were further categorized into high and low concentration levels for tissue-wise analysis. An analysis of variance (ANOVA) was conducted with uptake as the response variable and tissue, species, and time as independent variables, along with their interactions. Control data sets with suspected outliers were analysed using Rosner's test. For physiological parameters (quantum yield and reflectance index) the following analyses were performed: 1) a two-samples one-sided *t*-test to test for significant differences between treatments and controls for each species and exposure time; 2) an ANOVA, testing for the effects of nanoplastics on each physiological parameter. Finally, a two-samples one-sided *t*-test was performed to test significant differences in fresh *vs.* dry weights (indicating differences in tissue water content) of treated *versus* control trees for each tissue of each species and an ANOVA tested the shoot-to-root ratios at the end of the experiment for the two species.

## 3. Results and discussion

### 3.1. Nanoplastics are taken up by tree roots and transported to foliar tissues

**Fig. 1:**
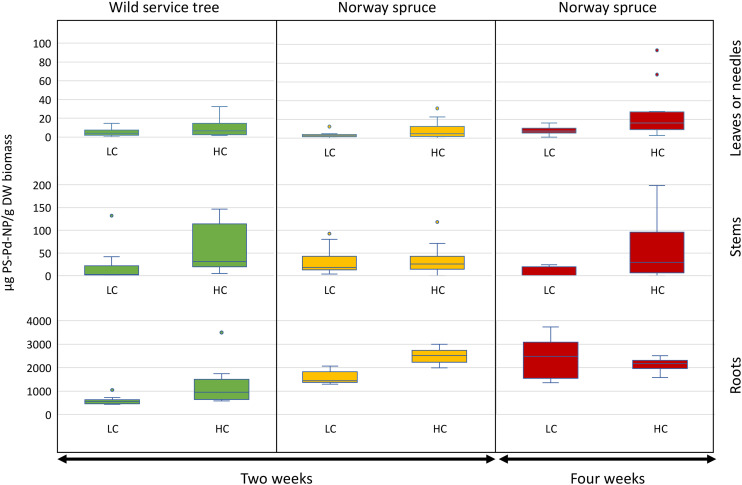
Box plots of the distribution of μg PS-Pd-NP per g DW biomass in the three different tissues (roots, stem, leaves or needles) of wild service tree and Norway spruce across the two different PS-Pd NP concentrations (10 and 30 mg L^−1^, LC and HC, respectively) and the two different exposure times (two and four weeks). Horizontal line: median, box: lower and upper quartile, whisker: standard deviation, dots indicate outliers (*n* = 12).

Nanoplastics were present in both roots and aboveground tree organs (stems and leaves or needles) at all PS-Pd-NP exposure concentrations and time points (Fig. 1). PS-Pd-NP concentrations associated with the roots were at least one order of magnitude higher than in aboveground tree organs for both wild service trees and Norway spruce. In most stems and leaves or needles for a given treatment (exposure duration, PS-Pd-NP concentration or tree species), concentrations of PS-Pd-NP per dry weight are not statistically different (Fig. 1 and Table S1†). Despite washing the roots prior to analysis, it is likely that the total root-associated NPs is a combination of both surface-adhered PS-Pd-NP and PS-Pd-NP internalized within the root. Nevertheless, as PS-Pd-NP were measured in aboveground tree organs of both species, this supports our hypothesis that transport from roots through the vascular system is occurring.

We had hypothesized that the less water conservative species wild service trees would take up NPs faster compared to xeromorphic species Norway spruce. However, when comparing the PS-Pd-NP concentration in the same organs of the two species after two weeks of exposure, there was a significantly higher concentration of PS-Pd-NP associated with the roots of Norway spruce (2512 ± 304 μg PS-Pd-NP per g of dry biomass) compared to those of wild service trees (1190 ± 823 μg PS-Pd-NP per g of dry biomass, *p* < 0.001, Fig. 1 and Table S2†). As root concentrations are assumed to be partially affected by externally adhered PS-Pd-NP, different root surface structures between the two species might play a larger role than water use strategies. We had, however, still expected higher transport to the aboveground organs of wild service trees, but there is no evidence of increased translocation for this species compared to the xeromorphic Norway spruce. Indeed, at two weeks, for both species and both PS-Pd-NP concentrations, the amount of PS-Pd-NP remained low in the above ground organs relative to the roots. For example, at the high concentration treatment, the wild service tree contained 58 ± 51 μg PS-Pd-NP per g of dry stem biomass and 10 ± 9 μg PS-Pd-NP per g of dry leaf biomass, while Norway spruce contained 35 ± 32 μg PS-Pd-NP per g of dry stem biomass and 8 ± 10 g PS-Pd-NP per g of dry needle biomass. We need to assume that at least over the short-term (*i.e.*, over a period of a few weeks as tested in our experiment) the water use strategy of tree species, and thus the amount of water taken up by the roots and transpired by the leaves, is not a decisive factor for transport of NPs to aboveground organs. If NPs transport is not coupled to water use, we have to consider that there is no complete apoplastic transport within the plant, but that the Casparian strip of the root endodermis might act as an efficient barrier that forces NPs to be taken up into the symplast.

There was overall little evidence that increasing the PS-Pd-NP concentration from 10 to 30 mg L^−1^ led to an increased uptake into plant organs. On one hand, there was strong evidence of increased uptake in Norway spruce roots after 2 weeks as a response to increase concentration (*p* < 0.001). On the other hand, increasing PS-Pd-NP concentrations only led to a moderate increase in uptake in wild service in Norway spruce needles after 4 weeks (*p* < 0.05). For all other treatments, no concentration effects were observed (Fig. 1 and Table S3†). This could be due to the relatively small differences in concentrations which were used in this experimental design. The concentrations selected in this study were used to complement and compare uptake and transport in previous work (*e.g.*, Del Real *et al.* 2022, where 10 and 30 mg L^−1^ PS NPs were used in hydroponic set-ups).^11^ While the experimental concentrations are higher than expected environmental concentrations,^43^ such concentrations were selected to be within the range of analytical measurement. Furthermore, it is anticipated that the ability for uptake and transport and the mechanisms involved are not depending on concentration.

The effect of longer exposure time for PS-Pd-NP uptake and impacts were investigated for Norway spruce. Here, strong evidence of increased uptake into roots after 4 weeks of exposure compared to 2 weeks was shown for low PS-Pd-NP concentration (*p* < 0.001). There was also evidence of increased uptake over time in the needles. Notably, we observed a moderate increase in uptake at high exposure concentrations (*p* < 0.05) (Fig. 1 and Table S4†).

In our study, we observed significant interactions between concentration–time and concentration–species in plant uptake dynamics (Fig. S4 and Table S5†). Stem association remained largely unaffected by treatments, hinting at their role as primary conduits for transporting substances to the leaves or needles, a concept that aligns well with physiological understanding. Root association exhibited a positive response to both high concentrations (*p* < 0.001) and prolonged exposure durations (*p* < 0.001) independently. However, when high PS-Pd-NP concentrations coincided with prolonged exposure, there was a decrease in root association (*p* < 0.001), suggesting a saturation effect wherein the roots' capacity for association may plateau under sustained NPs exposure. Notably, a strong interaction effect between high concentration and prolonged exposure was observed in needle association (*p* < 0.05), indicating a tendency for pollutant accumulation in needles under prolonged high NPs levels (Fig. S4 and Table S5†). These findings highlight the complex interplay of concentration and exposure duration in shaping NPs distribution within plant tissues, emphasizing the need for comprehensive assessment in environmental studies.

### 3.2. Physiological responses of trees when exposed to nanoplastics

**Fig. 2:**
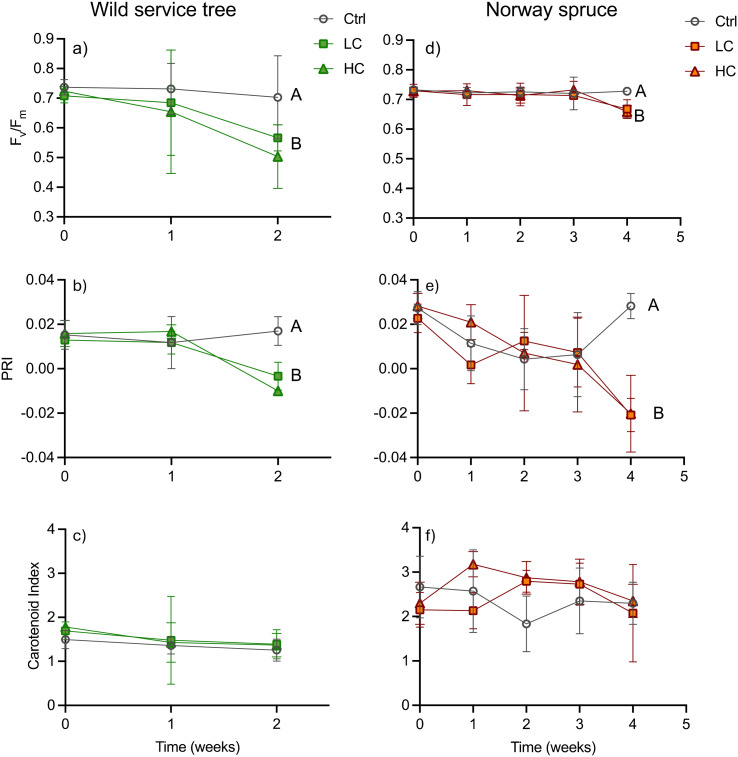
Potential quantum yield of photosystem II (*F*_v_/*F*_m_), photochemical reflectance index (PRI) and carotenoid index in the leaves of wild service trees (2a–c) and Norway spruce (2d–f) after different exposure times to 10 mg L^−1^ (LC) and 30 mg L^−1^ (HC) PS-Pd NP. Ctrl: untreated controls. Letters A and B indicate homogenous groups (and thus statistically significant differences at *p* < 0.05) in the ANOVA (with Tukey *post hoc* test). Data shown are mean values ± SD (*n* = 3–9).

The physiological response of both tree species to PS-Pd-NP were sublethal. Indeed, there were no observed effect of PS-Pd-NP on the dry weight or wet weight of tree biomass (Tables S6 and S7†), nor were there any significant differences in the root-to-shoot dried biomass ratios amongst controls or treated trees (Table S8†). Our observation of no effect of NPs on plant biomass is in agreement with a study in cress (*Lepidium sativum*).^12^ In this study only exposure to concentrations ≥50 mg (and thus higher than in our study) lead to biomass reduction and changes in root-to-shoot ratios. However, both low and high concentrations of PS-Pd-NP were deleterious on photosynthetic potential as indicated by chlorophyll fluorescence. In wild service trees the potential quantum yield of photosystem II (*F*_v_/*F*_m_) showed a tendency to decrease compared to the controls from week 1 in both NPs treatments, but the difference became significant only after two weeks (Fig. 2a). *F*_v_/*F*_m_ was then reduced by 19% and 28% in the LC and HC treatment, respectively. Thus, the HC treatment showed a tendency to lower *F*_v_/*F*_m_ but was not significantly different from the LC. In Norway spruce, *F*_v_/*F*_m_ stayed comparable to controls in both NPs treatments until week 3, and subsequently declined significantly (by 8% and 10% in the LC and HC treatment, respectively) in week 4 (Fig. 2d). The temporal patterns of *F*_v_/*F*_m_ matched those of PRI, as a proxy of the deep oxidation status of the xanthophyll cycle, in both treatments. In wild service trees, the PRI dropped in both the LC and the HC treatments in week 2 (Fig. 2b) from 0.017 in the control to −0.010 in the HC treatment, whereas it took until week 4 in Norway spruce until the PRI was lower in the NPs treatments (−0.020 in HC and LC) compared to the controls (0.028) (Fig. 2e). There were no significant differences in the carotenoid index (Fig. 2, panels c and f) between treatments and controls in both species. Furthermore, no significant differences were found for the NPCI, which is a proxy for the carotenoid to chlorophyll ratio (Fig. S5†). The PRI can be indicative for both xanthophyll de-epoxidation on short time scales as well as for a change in the carotenoid/chlorophyll ratio on longer time scales.^29^ Thus, the assessment of other reflectance indices for carotenoids helps to differentiate between short term xanthophyll and long-term carotenoid changes. Since neither the carotenoid index (as a measure for the leaf carotenoid content), nor the NCPI (as a measure for the carotenoid/chlorophyll ratio) were affected by the treatments, we have evidence to conclude that the change in PRI shows an increase in non-photochemical quenching of light energy *via* the xanthophyll cycle. The reduction of the photosynthetic capacity as observed in our study is in agreement with findings in *Eichhornia crassipes*.^14^ In this species, a strong reduction on net CO_2_ uptake was observed when plants were exposed to PS-Pd-NP.

On one hand, we could show that reduced quantum yield upon PS-Pd-NP exposure in both tree species examined here is related to increased de-epoxidation of the xanthophyll cycle pigments and thus increased quenching of light energy to heat. NPs are known to either directly destroy membranes by mechanical interaction or *via* induction of the production of reactive oxygen species.^44^ As mainly the electron transport of the photosynthetic light reaction is membrane bound, we might assume that the energy of excited chlorophyll molecules is less efficiently transferred to the electron transport chain and thus quenched *via* the xanthophyll cycle. On the other hand, our results indicate that wild service trees were negatively affected in their photosynthetic response earlier than Norway spruce despite there being no difference in leaf/needle PS-Pd-NP concentrations. For the roots, we postulated that the suberized cell walls of the endodermis, the Casparian strip, acts as an effective barrier against the transport of NPs. Needles, such as the ones of Norway spruce, also contain an endodermis with a functionality comparable to the roots,^45^ which is not present in leaves of broad-leaved species. The needle endodermis separates the vascular tissue from the mesophyll, where photosynthesis takes place. Even so, PS-Pd-NP concentrations in needles of Norway spruce and leaves of wild service trees were comparable, and so we might assume that the endodermis in the needles might have led to a compartmentation of PS-Pd-NP in the area of the vascular tissue, providing a longer protection of the photosynthetic tissues. Thus, we need to assume that not the difference in water use strategy but the anatomical differences between needles and leaves (of broadleaf trees) are responsible for the different temporal damage trajectories.

### 3.3. Contextualizing impacts of nanoplastics exposure, uptake, and transpiration on trees

In our study, NPs exposure and transport to the leaves had significant impact on photosynthetic capacity of both species. Despite similar NPs concentrations in the aboveground tree organs, there were different temporal impacts on tree physiology in the two species, which we attribute to anatomical differences between needles and leaves. While it has been shown that photosystem II efficiency (*i.e.*, *F*_v_/*F*_m_) is negatively affected by PS NPs in microalgae in a concentration dependent manner,^46,47^ not much is known about the impacts of these particles in higher plants. Roy *et al.* summarized the publications that dealt with the impact of NPs in general, and PS in particular, on plants, but the few results published to date are divergent.^48^ As an example, in barley, the antioxidative system as well as photosynthesis were strongly impaired by PS NPs at low temperatures,^49^ whereas in maize only the root metabolism was disturbed but photosynthesis remained unaffected.^19^ The negative response of photosynthesis under low temperature in barley can be reconciled with our observations. We could show that the drop in photosynthetic efficiency was related to an increase in non-photochemical quenching of light energy to heat *via* the xanthophyll cycle as indicated by a drop in PRI. Even though non-photochemical quenching reduces the efficiency of photosystem II, it also avoids photooxidative damage. The xanthophyll cycle is impeded at low temperatures^50^ and thus not efficient in protecting the photosystems. Thus, in particular photooxidation might have led to the strong impairment of photosynthesis and to an overloading of the antioxidative system in the NP-exposed barley. Maize in contrast to barley and the tree species used in our study operates with C_4_ photosynthesis.^51^ It is characterised by (a) CO_2_ preconcentration with the help of phosphoenolpyruvate carboxylase, which has a higher substrate affinity compared to the primarily CO_2_ fixing enzyme in C_3_ plants (ribulose-1,5-bisphosphate carboxylase oxygenase) and (b) by intercellular compartmentation of the preconcentration and final CO_2_ fixation step. Both, compartmentation and higher CO_2_ fixing efficiency might, thus, have led to a lower sensitivity of photosynthesis to NP exposure in maize. Our results showed that seedlings of forest trees show a systemic impairment of plant functioning as indicated by the photosynthetic efficiency and we have evidence that not water use strategies but rather anatomical differences between needles and leaves determines the sensitivity to NPs. Less photosynthetic carbon assimilation means less growth and lower investment in defence and storage.^52^ As carbon starvation due to an imbalance of assimilation and demand for maintenance metabolism is one important factor for tree mortality under drought,^53^ NPs pollution might thus contribute to the negative effects of global climate change on trees and forests. Together with air pollution, novel pests and diseases, and climate change, NPs might thus add to the stressors forest trees will be exposed to in future.

## 4. Conclusions

Using a hydroponic growth chamber, we have shown that PS-Pd-NP uptake into trees depends on exposure concentration and duration, rather than the trees' water use strategy. Furthermore, PS-Pd-NP were translocated from the roots into aboveground tree organs (stems, leaves or needles), which ultimately changed certain physiological functioning. In particular, photosynthetic efficiency decreased upon PS-Pd-NP exposure where the broadleaf species showed effects sooner than the needle-leaf species, possibly due to NPs compartmentation within needles facilitated by the endodermis. Our study has shed light on the intricate relationship between concentration–time and concentration–species in shaping plant uptake dynamics. Our main finding underscores the significance of interaction between concentration–time and concentration–species on PS-Pd-NP uptake. The observation that stem association remains unaffected by treatments highlights its critical role as a primary conduit for transporting NPs to leaves or needles.

Given the likelihood of trees facing multiple, concurrent stressors from anthropogenic pollution and climate change, including NPs, it is crucial to consider the cumulative effects on vegetation. While our findings suggest that the impacts of NPs pollution on trees can vary based on factors such as tree physiology, needle morphology, and water use strategies, further research is needed to fully understand the relative and cumulative negative effects of NPs in different environmental contexts. Nevertheless, our study offers insights into the potential risks of NPs pollution on trees and highlights the importance of mapping NPs hot-spots (*e.g.*, urban centers, *etc.*) and understanding the susceptibility of different tree species in various locations. By doing so, we can better assess and mitigate the risks posed by NPs pollution on tree health and ecosystem functioning.

## Data availability

The main manuscript contains two figures, including uptake of NPs into tree tissue and tree physiological responses to NPs exposure. The data has also been made publicly available at: https://doi.org/10.5281/zenodo.11526158.

## Conflicts of interest

There are no conflicts to declare.

## Supplementary Material

EN-011-D4EN00286E-s001
