# Supplementary material for: Uptake and physiological impacts of nanoplastics in trees with divergent water use strategies
Source: Environ Sci Nano. 2024 Jul 11;11(8):3574–84. doi: 10.1039/d4en00286e (PMC11309015; doi:10.1039/d4en00286e)
Supplement: EN-011-D4EN00286E-s001 [file EN-011-D4EN00286E-s001.pdf]

Electronic Supplementary Material (ESI) for Environmental Science: Nano.
This journal is © The Royal Society of Chemistry 2024

Supplemental Information

Uptake and physiological impacts of nanoplastics in trees with divergent water use strategies

Maria Elvira Murazzi[1,2], Alice Pradel[2], Roman B. Schefer[2], Arthur Gessler[1,2], Denise M. Mitrano[2*]

1. WSL Swiss Federal Institute for Forest, Snow and Landscape Research, Zürcherstrasse 111, 8903 Birmensdorf, Switzerland
2. Department of Environmental Systems Science, ETH Zurich, Universitatstrasse 16, 8092 Zurich, Switzerland

*Corresponding author: denise.mitrano@usys.ethz.ch

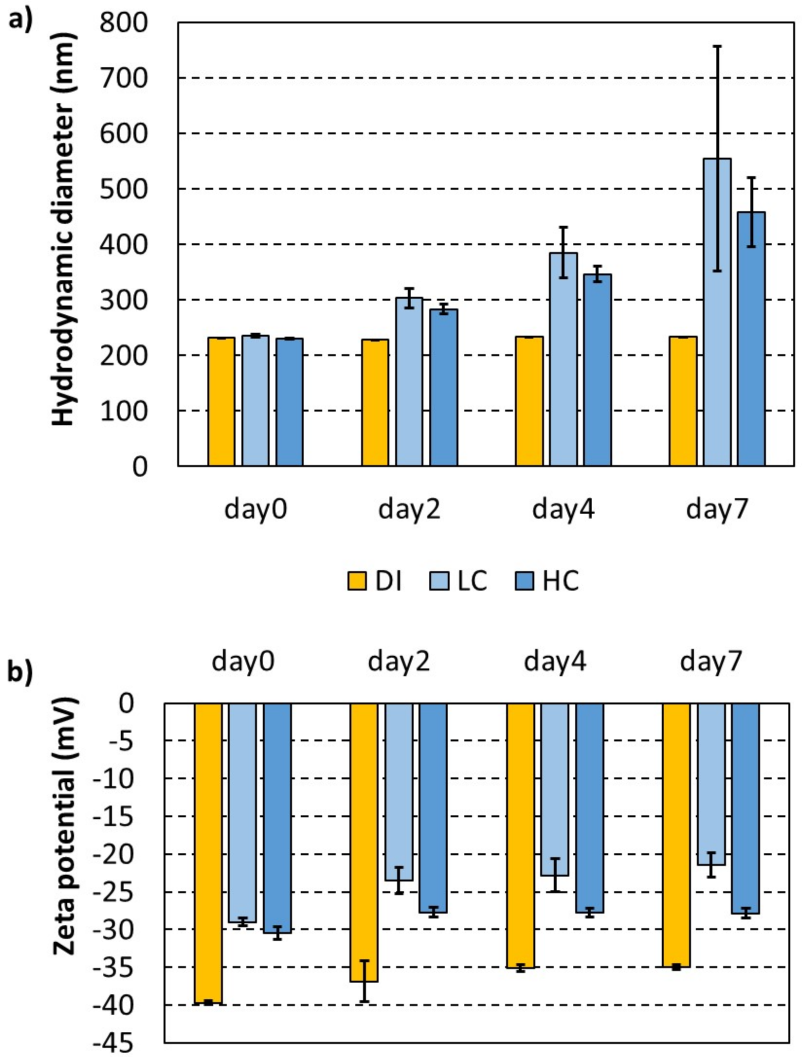

Figure S1. Evolution of the a) hydrodynamic diameter (nm) and b) zeta potential (mV) of the model Palladium doped polystyrene nanoplastics (PS-Pd-NP) in deionized water (DI) at high particle concentration (30 mg/L) and growth media at low (LC) and high (HC) concentrations (10 and 30 mg/L, respectively) over one week. These time points correspond to the time at which solutions were renewed during the exposure experiments. Error bars represent standard deviation.

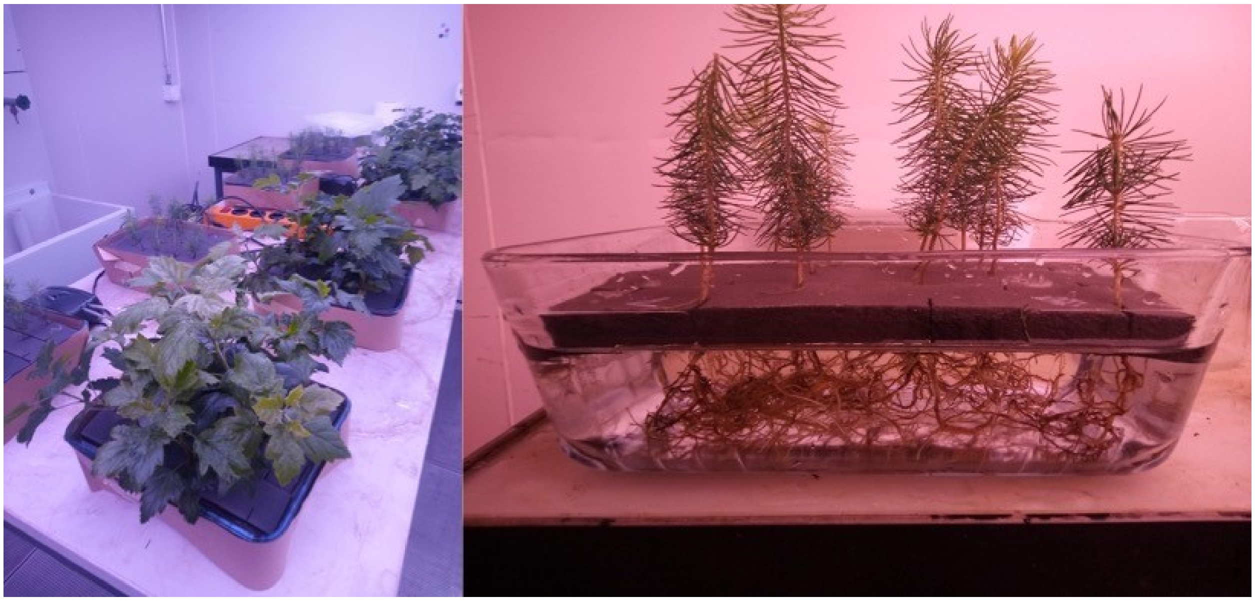

Figure S2: Photos of experiment set-up where 12 trees of each species were grown in a hydroponic system. The root crown of each tree was inserted into the floating mat through a vertical slot. In this way, only the roots were submerged in the liquid solution, whereas the aboveground tissues remained physically isolated from the nutrient solution and potential nanoplastics contamination.

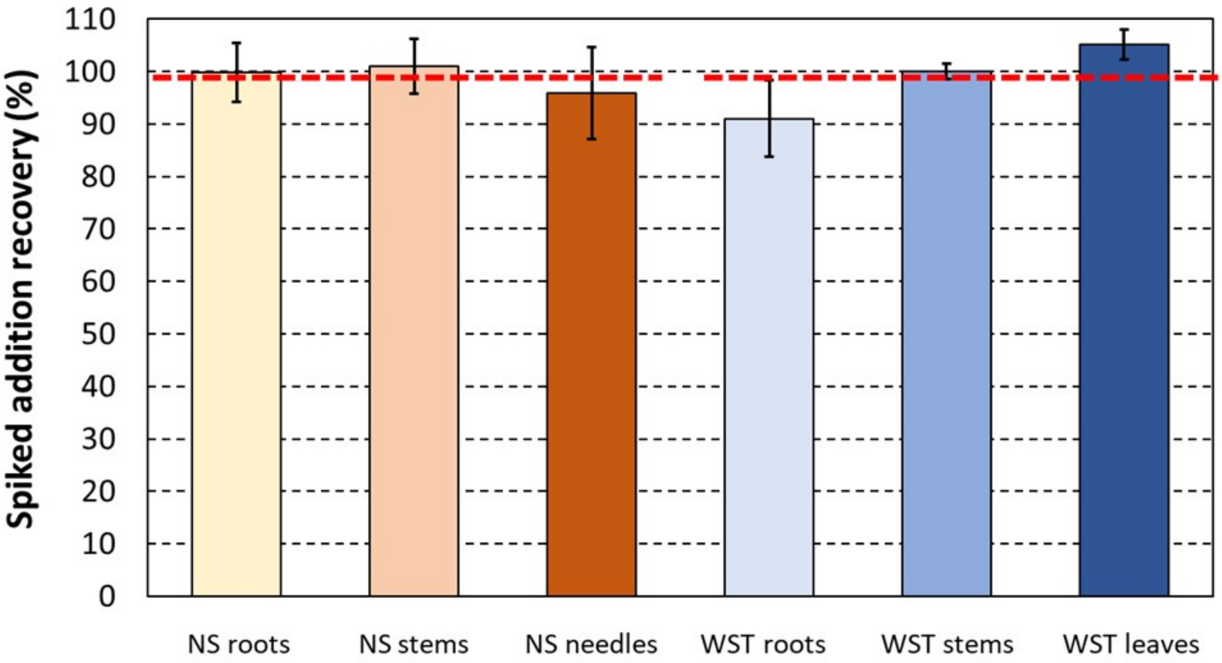

Figure S3. Average nanoplastics spiked addition recovery (%) of PS-Pd-NP dispersions (1 µg/L, 2.5 µg/L, and 5 µg/L) on the different tissues of Norway spruce (NS) and wild service tree (WST) (n=3). Results from all concentrations for each tree organ are graphed together, and error bars represent standard deviations. The red line indicates the average recoveries across all tree organs.

Table S1: p-values of the ANOVA test comparing comparing the NPs uptake between the three tissues groups within one species. Significance levels: *** $p<0.001$, ** $p<0.01$, * $p<0.05$.

	**Low Concentration (LC)**								
	***S. torminalis* (2 weeks)**			***P. abies* (2 weeks)**			***P. abies* (4 weeks)**		
	**Est.**	**p-value**	**Sig.**	**Est.**	**p-value**	**Sig.**	**Est.**	**p-value**	**Sig.**
**Roots vs Leaves**	585.64	<0.001	***	1577.11	<0.001	***	2419.83	<0.001	***
**Roots vs Stems**	572.26	<0.001	***	1549.42	<0.001	***	2420.24	<0.001	***
**Stems vs Leaves**	13.37	0.580		27.69	0.014	*	0.40	1.000	
	**High concentration (HC)**								
	***S. torminalis* (2 weeks)**			***P. abies* (2 weeks)**			***P. abies* (4 weeks)**		
	**Est.**	**p-value**	**Sig.**	**Est.**	**p-value**	**Sig.**	**Est.**	**p-value**	**Sig.**
**Roots vs Leaves**	1179.55	<0.001	***	2503.56	<0.001	***	2101.55	<0.001	***
**Roots vs Stems**	1131.63	<0.001	***	2477.30	<0.001	***	2077.28	<0.001	***
**Stems vs Leaves**	47.91	0.026	*	26.26	0.509		24.27	0.594	

Table S2: P-values of the linear model comparing the NPs uptake at 2 weeks in *Picea abies* vs. *S. torminalis*. Significance levels: *** $p<0.001$, ** $p<0.01$, * $p<0.05$.

	***S. torminalis* vs. *P. abies* (2 weeks)**			
**Tissue**	**Concentration**	**Effect**	**p-value**	**Significance**
**Leaves**	Low	2.11	0.999	
	High	1.70	0.999	
**Stems**	Low	-12.20	0.995	
	High	23.36	0.840	
**Roots**	Low	-989.35	<0.001	***
	High	-1322.30	<0.001	***

Table S3: P-values of the linear model comparing the effects of NPs concentration on uptake. Significance levels: *** $p<0.001$, ** $p<0.01$, * $p<0.05$.

**Low vs. High Concentration**			
***S. torminalis* (2 weeks)**			
**Tissues**	**Estimate**	**p-value**	**Significance**
**Leaves**	4.61	0.985	
**Stems**	39.15	0.261	
**Roots**	598.52	0.114	
***P. abies* (2 weeks)**			
**Tissues**	**Estimate**	**p-value**	**Significance**
**Leaves**	5.02	0.977	
**Stems**	3.58	0.999	
**Roots**	931.47	<0.001	**
***P. abies* (4 weeks)**			
**Tissues**	**Estimate**	**p-value**	**Significance**
**Leaves**	18.26	0.016	*
**Stems**	42.94	0.165	
**Roots**	-300.02	0.853	

Table S4: Interaction plots for the effect of concentration and time on predicted NPs uptake for leaves, stems and roots. Significance levels: *** $p<0.001$, ** $p<0.01$, * $p<0.05$.

	**2 weeks vs. 4 weeks (*P. abies*)**			
**Tissue**	**Concentration**	**Estimate**	**p-value**	**Significance**
**Needles**	Low	4.85	0.848	
	High	18.09	0.020	*
**Stems**	Low	-23.24	0.432	
	High	16.11	0.717	
**Roots**	Low	847.57	<0.001	***
	High	-383.91	0.228	

Table S5: Estimates and p-values of the linear model for the effects of concentration, time, and species on NPs uptake in leaves, stems and roots. Interactions between concentration-time and concentration-species were included. Significance levels: *** $p<0.001$, ** $p<0.01$, * $p<0.05$.

	**Individual and Interaction Effects**			
**Tissue**	**Interaction**	**Estimate**	**p-value**	**Significance**
**Leaves**	Intercept	3.36	0.361	
	High vs. Low	5.02	0.335	
	4-weeks vs. 2-weeks	4.85	0.352	
	ST vs. PA	2.11	0.684	
	high & 4-weeks vs. low & 2-weeks	13.24	0.045	*
	high & ST vs. low & PA	-0.41	0.956	
**Stems**	Intercept	31.06	0.009	**
	High vs. Low	3.59	0.827	
	4-weeks vs. 2-weeks	-23.25	0.160	
	ST vs. PA	-12.21	0.458	
	high & 4-weeks vs. low & 2-weeks	39.35	0.094	
	high & ST vs. low & PA	35.57	0.129	
**Roots**	Intercept	1580.47	<0.001	***
	High vs. Low	931.47	<0.001	***
	4-weeks vs. 2-weeks	847.57	<0.001	***
	ST vs. PA	-989.35	<0.001	***
	high & 4-weeks vs. low & 2-weeks	-1231.49	<0.001	***
	high & ST vs. low & PA	-332.94	0.275	

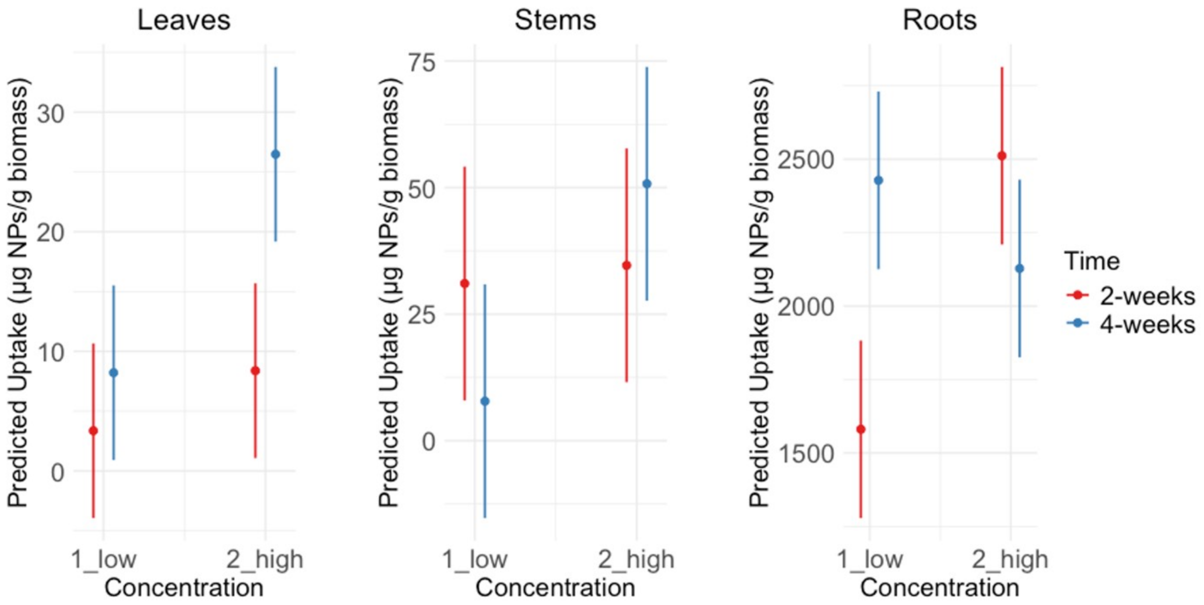

Figure S4: Interaction plots for the effect of concentration and time on NPs uptake for leaves, stems and roots.

Table S6: Dry weight (g) of wild service tree and Norway spruce at the end of the experiments. Data shown are mean values ± SD.

	**Wild service tree 2 weeks**		
	**Control**	**LC**	**HC**
**Leaves**	1 ± 0.39	1.19 ± 0.26	1.08 ± 0.36
**Stems**	0.42 ± 0.22	0.38 ± 0.16	0.37 ± 0.19
**Roots**	0.31 ± 0.1	0.31 ± 0.08	0.31 ± 0.17
	**Norway spruce 2 weeks**		
	**Control**	**LC**	**HC**
**Needles**	0.33 ± 0.07	0.4 ± 0.13	0.37 ± 0.09
**Stems**	0.13 ± 0.04	0.17 ± 0.05	0.13 ± 0.06
**Roots**	0.17 ± 0.06	0.22 ± 0.07	0.2 ± 0.08
	**Norway spruce 4 weeks**		
	**Control**	**LC**	**HC**
**Needles**	0.18 ± 0.06	0.2 ± 0.07	0.18 ± 0.06
**Stems**	0.07 ± 0.02	0.07 ± 0.02	0.07 ± 0.03
**Roots**	0.13 ± 0.05	0.13 ± 0.04	0.11 ± 0.06

Table S7: Water content (%) in the three different tissues for each species and each exposure time calculated as 100% - 100 (Dry weight / Fresh weight) Data shown are mean values ± SD.

	**Wild service tree 2 weeks**		
	**Control**	**LC**	**HC**
**Leaves**	22.71 ± 14.25	13.58 ± 7.17	14.99 ± 12.76
**Stems**	49.59 ± 9.07	47.36 ± 12.71	51.56 ± 9.73
**Roots**	77.12 ± 3.04	76.55 ± 3.54	77.23 ± 2.93
	**Norway spruce 2 weeks**		
	**Control**	**LC**	**HC**
**Needles**	67.04 ± 3.46	67.27 ± 2.74	66.95 ± 1.8
**Stems**	66.56 ± 4.22	64.41 ± 2.82	68.26 ± 2.84
**Roots**	78.73 ± 2.19	79.39 ± 2.9	78.86 ± 2.07
	**Norway spruce 4 weeks**		
	**Control**	**LC**	**HC**
**Needles**	64.35 ± 13.77	62.74 ± 18.79	59.94 ± 15.57
**Stems**	64.2 ± 10.08	59.1 ± 9.3	60.1 ± 20.3
**Roots**	83.57 ± 2.71	81.25 ± 3.36	84.97 ± 3.84

Table S8: Dry weight shoot-to-root ratios for wild service tree and Norway spruce at the end of the experiments. Data shown are mean values ± SD. F-ratios (the ratio of the between group variance to the within group variance) and p-values were assessed by one-way ANOVA.

**Species, duration**	**Treatment**			**F-ratio**	**p-value**
	**C**	**LC**	**HC**		
Wild service tree (2 weeks)	4.76 ± 1.58	5.41 ± 1.69	5.35 ± 1.90	0.808	0.452
Norway spruce (2 weeks)	2.17 ± 0.48	2.09 ± 0.51	2.02 ± 0.47	0.264	0.769
Norway spruce (4 weeks)	2.13 ± 0.81	2.21 ± 0.82	2.55 ± 1.13	0.889	0.418

There were no significant differences in shoot-to-root ratios between the controls and the two treatments for both species. For wild service tree there was a slight, though not significant, tendency for a higher shoot-root ratio in both LC and HC exposure concentrations after two weeks whereas after the same incubation time such trends were not visible in Norway spruce. However, some tendency for increased shoot-root ratios in HC were visible for Norway spruce after four weeks. Two- and four-weeks incubation may not be sufficient to induce clear changes in growth patterns, even in seedlings with their relatively high growth rates. The slight tendency we observed for increased shoot-root ratios in wild service tree after two and in Norway spruce after four weeks might indicate relatively reduced C allocation to the roots, which is known to occur when plants are exposed to stressors (Joseph et al., 2020).

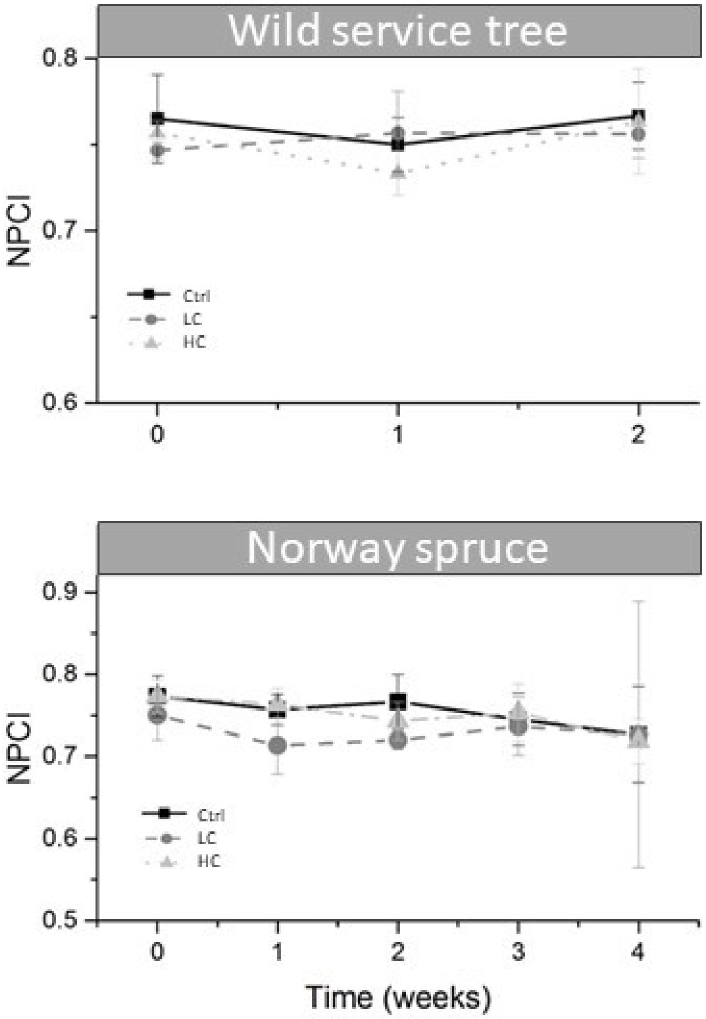

Figure S5: Normalized Pigment Chlorophyll Index (NPCI) of wild service tree and Norway spruce after different exposure times to low (LC) and high concentrations (HC) of plastic nanoparticles. Ctrl: untreated controls. Data shown are mean values (N= 3-9) ± SD.

References

1. Joseph, J.; Gao, D.; Backes, B.; Bloch, C.; Brunner, I.; Gleixner, G.; Haeni, M.; Hartmann, H.; Hoch, G.; Hug, C.; Kahmen, A.; Lehmann, M.M.; Li, M-H.; Luster, J.; Peter, M.; Poll, C.; Rigling, A.; Rissanen, K.A.; Ruehr, N.K.; Saurer, M.; Schaub, M.; Schönbeck, L.; Stern, B.; Thomas, F.M.; Werner, R.A.; Werner, W.; Wohlgemuth, T.; Hagedorn, F.; Gessler, A., Rhizosphere activity in an old-growth forest reacts rapidly to changes in soil moisture and shapes whole-tree carbon allocation. *Proceedings of the National Academy of Sciences* **2020**, 117: 24885-24892.
